# Tracing the Origin of the East-West Population Admixture in the Altai Region (Central Asia)

**DOI:** 10.1371/journal.pone.0048904

**Published:** 2012-11-09

**Authors:** Mercedes González-Ruiz, Cristina Santos, Xavier Jordana, Marc Simón, Carles Lalueza-Fox, Elena Gigli, Maria Pilar Aluja, Assumpció Malgosa

**Affiliations:** 1 Unitat d’Antropologia Biològica, Dept. BABVE, Universitat Autònoma de Barcelona, Bellaterra, Barcelona, Spain; 2 Institut Català de Paleontologia Miquel Crusafont (ICP), Universitat Autònoma de Barcelona, Bellaterra, Barcelona, Spain; 3 Institut de Biologia Evolutiva, CSIC-UPF, Barcelona, Spain; University of Florence, Italy

## Abstract

A recent discovery of Iron Age burials (Pazyryk culture) in the Altai Mountains of Mongolia may shed light on the mode and tempo of the generation of the current genetic east-west population admixture in Central Asia. Studies on ancient mitochondrial DNA of this region suggest that the Altai Mountains played the role of a geographical barrier between West and East Eurasian lineages until the beginning of the Iron Age. After the 7th century BC, coinciding with Scythian expansion across the Eurasian steppes, a gradual influx of East Eurasian sequences in Western steppes is detected. However, the underlying events behind the genetic admixture in Altai during the Iron Age are still unresolved: 1) whether it was a result of migratory events (eastward firstly, westward secondly), or 2) whether it was a result of a local demographic expansion in a ‘contact zone’ between European and East Asian people. In the present work, we analyzed the mitochondrial DNA lineages in human remains from Bronze and Iron Age burials of Mongolian Altai. Here we present support to the hypothesis that the gene pool of Iron Age inhabitants of Mongolian Altai was similar to that of western Iron Age Altaians (Russia and Kazakhstan). Thus, this people not only shared the same culture (Pazyryk), but also shared the same genetic east-west population admixture. In turn, Pazyryks appear to have a similar gene pool that current Altaians. Our results further show that Iron Age Altaians displayed mitochondrial lineages already present around Altai region before the Iron Age. This would provide support for a demographic expansion of local people of Altai instead of westward or eastward migratory events, as the demographic event behind the high population genetic admixture and diversity in Central Asia.

## Introduction

Historically, Central Asia has been a crossroad between West and East Eurasian people leading to the current high population genetic admixture and diversity. The origin of this diversity may go back as early as the Iron Age, more than two thousand years ago, with the dispersal of mounted pastoral nomads across the Eurasian steppes [1], [2], [3]. The present study deals with early contacts between West and East Eurasian populations and specifically those that occurred in the Altai region (Central Asia). Because the Altai Mountains represent a natural boundary between West and East Eurasian steppes, this region is key to understanding demographic events in the steppes of Central Asia.

**Figure 1:**
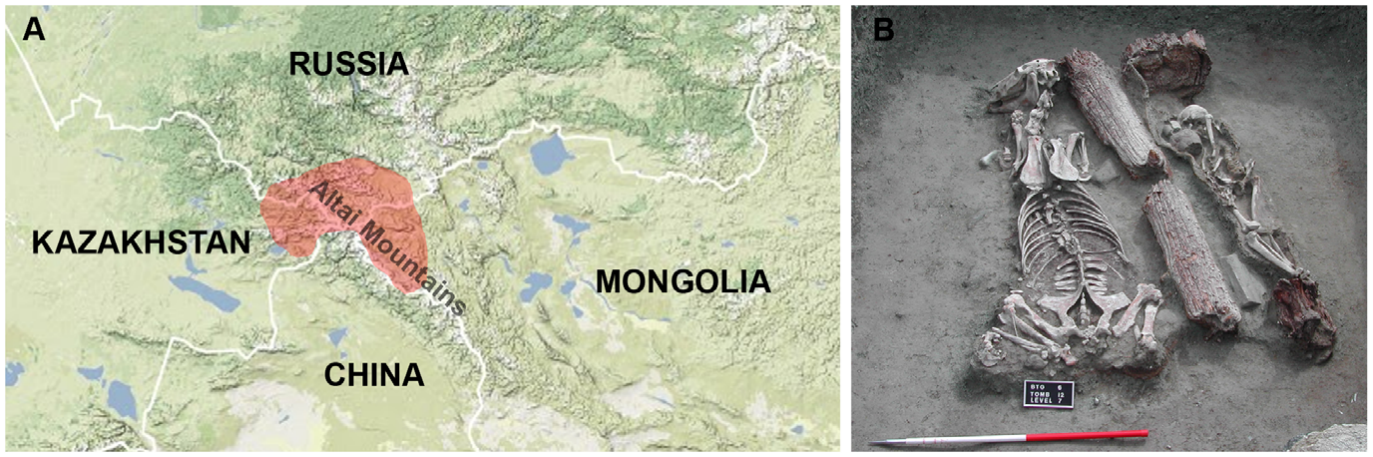
Pazyryk burials from the Altai Mountains. A. Geographical location of Pazyryk culture sites in the Altai regions of South Siberia, Kazakhstan and Western Mongolia. B. Pazyryk burial from Baga Turgen Gol site, Bayan-Ölgiy province, Western Mongolia.

Archaeological work conducted by a Spanish–French–Mongolian team in the Mongolian Altai during the period 2005–2007 discovered burial sites belonging to the Pazyryk culture. This was the first time that this culture was found in Mongolia [4], [5] Pazyryk is the name given to Iron Age nomadic tribes who inhabited the high steppes of the Altai Mountains between the fifth and third centuries BC. This culture is known from the discoveries of stone tumuli holding frozen bodies of warriors buried with their horses and their weapons [6], [7]. Pazyryk culture sites were first described in the Altai region of South Siberia and East Kazakhstan [6], [7], [8], [9], [10]. More recently, two different expeditions [5], [11] also discovered Pazyryk burials in the Mongolian Altai, indicating that this Iron Age people had also spread into East Asia (Fig. 1).

The Pazyryks have traditionally been associated with the Eastern Scythians. Scythians, whose history is well-known from the ancient texts of Herodotus (484–425 BC), was the name that the Greeks gave to a number of separate Indo-European-speaking nomadic groups living in the region encompassing the Pontic-Caspian steppe (in Eastern Europe) and the steppe of Central Asia. The Scythian culture (7th-2nd century BC) flourished in this region from local Indo-European peoples that emerged at the Pontic-Caspian steppe in about 2000BC and expanded eastward until they reached the Altai Mountains. Advances in technology that favoured mounted nomadic pastoralism were the triggers for the expansion of Scythian culture across the Western Eurasian steppe. The end of Scythian period might be related with the westward migrations of nomadic Turkomongolian tribes coming from East Asia since the 3rd century BC, which marked the end of Indo-European domination of the steppe [12], [13], [14], [15].

Archaeological findings, almost entirely provided by burial site discoveries, documented that the Scythians had European morphological features [7], [8], [12]. However, several recent works focusing on ancient mitochondrial DNA (mtDNA) of Eastern Scythian burials [9], [10], [11], [16], [17], [18], [19] revealed that this population has a mixed mtDNA composition of West and East Eurasian lineages. This is particularly interesting for the timing of the early contacts between European and Asian people in Altai because all ancient DNA samples analysed so far from Central Asia belonging to a period before the Iron Age bore West Eurasian lineages [18], [20].

These molecular data raise two likely hypotheses for the origin of the genetic diversity and admixture among the Iron Age inhabitants of the Altai: 1) people holding west Eurasian lineages arrived at Altai Mountains with the eastward migration of Scythians and, once settled, they began to establish relationships with the neighbouring communities from East Asia holding East Eurasian lineages; 2) this was the result of the admixture between the native people inhabiting either sides of the Altai Mountains (people with West Eurasian lineages in Western Altai and East Eurasian lineages in the Eastern Altai), as a result of a demographic expansion during the Scythian period. Hence, the second hypothesis would provide support to the cultural transmission against the demic diffusion during the Scythian period.

The skeletal remains unearthed by our team from Bronze and Iron Age tumuli in the Mongolian Altai offer a unique opportunity to get further insights into the ancient Altaians. Here, we aim to shed light on the origin of the current east-west population admixture in Central Asia, specifically in the Altai region, by analyzing the mitochondrial DNA lineages of these skeletons. Owing to the clear geographical structuring of the East and West Eurasian lineages in Central Asia population, mtDNA is particularly suited for the study of admixture in this region [18]. This study may contribute to providing early evidence of population admixture between European and East Asian people, as well as the underlying causes behind this demographic event in the Eurasian steppes.

## Materials and Methods

### Samples and DNA Extraction

Skeletal remains from 19 individuals of Bronze and Iron Age [5] were retrieved from four archaeological sites located in Bayan-Ölgiy province (Mongolia, Altai) (Fig. 1, Table 1). For the nineteen exhumed individuals, teeth and bone samples were taken in the field by one of us (XJ) following sterility criteria and were stored in cold conditions. Afterwards, samples were taken to the laboratory dedicated to paleogenetic studies at the Universitat Autònoma de Barcelona where they were processed. Independent replications were performed at the Institut de Biologia Evolutiva (CSIC-UPF).

**Table 1:** Sample distribution by Mongolian Altai sites and period.

				Sex		
Period	Site	Individual	Age	Morphological	Amelogenin	SRY
Bronze Age	Alag Erdene	AE05.T2	Adult	IND	♂	NR
		AE05.T3	Adult	IND	♂	NR
	Tsengel Khairkhan	TSA07.T4	Adult	♂	♂	♂
Iron Age, Pazyryk Culture	Baga Turgen Gol	BTG05.T1	Adult	IND	NR	NR
		BTG05.T2	Adult	IND	♂	NR
		BTG05.T8.1	Adult	♂	♂	NR
		BTG05.T8.2	Adult	♀	♀	NR
		BTG05.T8.3	Subadult	IND	NR	NR
		BTG06.T3	Adult	♂	♂	NR
		BTG06.T8	Adult	♂	NR	NR
		BTG06.T10A	Subadult	IND	♂	♂
		BTG06.T10B	Adult	♀	♂	NR
		BTG06.T11A	Adult	♂	♂	NR
		BTG06.T11B	Adult	♂	NR	NR
		BTG06.T12	Adult	♂	♂	NR
		BTG06.T13	Adult	♂	♀	♂
	Tsengel Khairkhan	TSK07.T1	Adult	♂	♂	NR
		TSK07.T2A	Subadult	IND	NR	NR
		TSK07. T2B	Adult	♂	♂	♂

IND- Indeterminate.

NR- No results.

For DNA extraction, 0.1 g of powder was extracted from teeth pulp cavities; when bones were used, 0.5 g of powder was collected from the internal compact tissue. After DNA treatment and extraction (as described in [21]), purification of the samples was performed with a JetQuick PCR Purification kit (Genomed Löhne, Germany) to remove any possible inhibitors that the samples might carry and it was stored at 4°C [22].

**Table 2:** Results of HVRI sequencing and PCR-RFLP of coding region informative polymorphisms.

		Sequence HVRI		PCR-RFLPs										
Sample		16051–16400	HaplogroupHVRI	Hae III +663	Hind II −13259	Alu I−5176	Hae II+4830	Nla III−4577	Bst 0I −13704	Alu I −7025	Hae II −9052	Hinf I +12308	Alu I +15606	Haplogroup PCR-RFLPs
Bronze	AE05.T2	223, 292, 362	D	−	+	−								D
	AE05.T3	192, 223, 295, 362	D	−	+	−								D
	TSA07.T4*	092, 223, 311, 362	D			−								D
Iron	BTG05.T1	223, 319, 362	D	−	+	−								D
	BTG05.T2	093, 224, 311, 319	K	−	+	+					−	−	+	K
	BTG05.T8.1	129, 256, 270, 304, 399	U5a1		+							+		U
	BTG05.T8.2	223, 298, 327	C		−	+								C
	BTG05.T8.3**	223, 239, 243, 319, 362	D											
	BTG06.T3	069, 126	J					+	−			−		J
	BTG06.T8*	223, 239, 243, 319, 362	D	−		−								D
	BTG06.T10A	172, 311	HV6	−		+		+		+	+			HV
	BTG06.T10B	223, 274, 311, 362	D			−								D
	BTG06.T11A	093, 224, 311, 319	K				−				−	−	+	K
	BTG06.T11B*	093, 224, 311, 319	K								−	−	+	K
	BTG06.T12	192, 256, 270, 304, 399	U5a1									+		U
	BTG06.T13	093, 129, 223, 298, 327, 362	C		−									C
	TSK07.T1*	093, 223, 242, 278, 290, 311, 319	A	+		+								A
	TSK07.T2A	223, 227, 278, 362	G2a			+	+					−		G
	TSK07.T2B	126, 163, 186, 189, 294	T1								+		−	T

* Samples replicated in Institut de Biologia Evolutiva.

** Sample amplified in Institut de Biologia Evolutiva.

Haplogroup attribution based on HVRI and in PCR-RFLPs are also presented for each sample. Variant positions from HVRI are shown between 16051 to 16400 minus 16000.

### Mitochondrial DNA Analysis

For each sample, the mtDNA hypervariable region I (HVRI) was amplified and sequenced, and coding region informative polymorphisms for haplogroup assignment were analysed by PCR-RFLPs. The PCR reactions were carried out in a final volume of 50 µl and Taq polymerase (Bioline®) was used. Each PCR reaction consisted of an initial denaturation step (5 min at 94°C) followed by 39 cycles of PCR (50 s at 94°C, 1 min at annealing temperature depending on the region to be amplified, and 1 min at 72°C) and a final extension step of 5 min at 72°C, or of 10 min if the amplified segment was to be cloned. Amplified fragments were then visualized with Ethidium Bromide staining in a 3% agarose gel [23]. To analyse the HVRI, four overlapping fragments were used (Table S1). These were subsequently sequenced and cloned. Sequence reactions were carried out using the sequencing kit BigDye Terminator v.3.1 (Applied Biosystems, Carslbad, USA) according to the manufacturer’s specifications and run in an ABI 3130XL sequencer.

**Figure 2:**
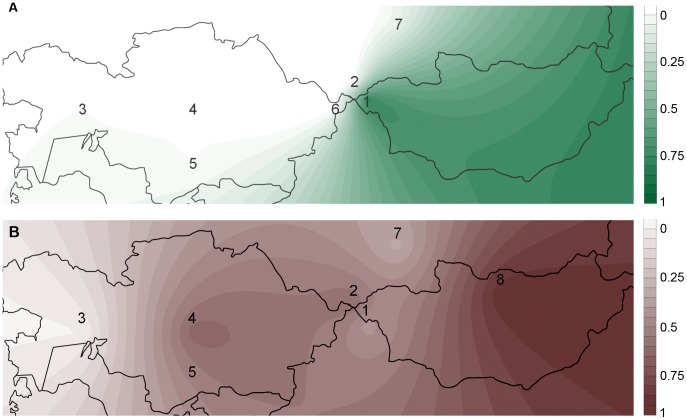
Spatial frequency distribution maps of East Eurasian lineages. A- Pre-Iron Age period; B- Iron Age period. Frequency values and detailed information for populations 1–8 are shown in table 3. 1- Mongolia (Altai), 2- Gorny Altai, 3- West Kazakhstan, 4- Central Kazakhstan, 5- South Kazakhstan, 6- East Kazakhstan, 7- SW Siberia, 8- Mongolia (Egyin Gol).

For all samples, the fragment containing the majority of HVRI mutations was cloned using the Topo TA Cloning® kit (Invitrogen, Carslbad, USA) following the manufacturer’s instructions. The colonies were harvested and subjected to PCR with M13 universal primers; for each sample 10 inserts of the right size were subsequently sequenced.

**Table 3:** Frequency of West and East Eurasian haplogroups in ancient Eurasian populations prior to Iron Age and from the Iron Age.

					% Haplogroups						
Period		Geographic zone		Number of samples		West Eurasian		East Eurasian		References	
Bronze Age	Mongolia (Altai)		3		0		100		Present study		
Neolithic and Bronze Age		Gorny Altai		4		100		0		[3]	
Bronze Age		Kazakhstan		13		100		0		[7]	
		West		3		100		0			
		Central		1		100		0			
		South		2		100		0			
		East^#^		7		100		0			
Bronze Age		SW Siberia		11		90.9		9.1		[4]	
Iron Age, Pazyryk		Altai Mongolia		19		57.9		42.1		Present study; [2]	
Iron Age, Pazyryk	Republic of Altai/Gorny Altai		10		40		60		[3], [5], [6]		
Iron Age		Kazakhstan		13		53.8		46.2		[7]	
		West		3		100		0			
		Central		8		37.5		62.5*			
	South		2		50		50*				
Iron Age	SW Siberia		15		66.7		33.3		[4]		
Late Iron Age		Mongolia (Egyin Gol)		46		10.9		89.1*		[1]	

#includes 5 samples Bronze/Iron Age (8th to 7th BC).

* Included one probable South Asia (Indian) lineage.

For coding region analysis 10 coding region segments, determining the 10 Eurasian haplogroups, were analysed by PCR-RFLPs. Restriction sites and the primers used to amplify each specific fragment of the coding region are shown in Table S1.

**Table 4:** mtDNA HVRI diversity (from nucleotide positions 16051 to 16400) in Pazyryk population and populations used for comparison.

POPULATION	N	*K (K%)*	*S (S%)*	*Ĥ ± sd*	*π ± sd*
*PAZMG2*	3	2 (66.66)	8 (2.28)	0.667±0.314	0.0189±0.015
*BRNRA*	20	8 (40.00)	14 (3.98)	0.732±0.094	0.0108±0.006
*LAJ*	14	8 (57.15)	13 (3.70)	0.901±0.058	0.0117±0.007
*KZBR*	13	10 (76.92)	18 (5.12)	0.923±0.069	0.0144±0.008
TUB	11	9 (81.81)	23 (6.55)	0.945±0.066	0.0180±0.010
TUV	63	36 (57.14)	52 (14.80)	0.959±0.013	0.0208±0.011
GEOR	45	28 (62.22)	41 (11.70)	0.964±0.016	0.0152±0.008
SIB	515	120 (23.30)	90 (25.65)	0.966±0.003	0.0222±0.001
*PAZMG1* *	16	13 (81.25)	28 (7.99)	0.967±0.036	0.023±0.012
*EGOL*	46	26 (56.52)	38 (10.82)	0.968±0.012	0.0183±0.010
KGEO	29	22 (75.86)	41 (11.68)	0.968±0.028	0.0140±0.008
BUR	33	25 (75.75)	52 (14.81)	0.974±0.012	0.0199±0.011
*PAZRA*	10	9 (90.00)	25 (7.12)	0.978±0.054	0.0079±0.006
TURK	74	64 (86.50)	77 (21.93)	0.994±0.004	0.019±0.010
*SBIR*	15	13 (86.66)	30 (8.55)	0.981±0.031	0.0232±0.013
*KZIR*	13	12 (92.30)	16 (4.55)	0.982±0.035	0.0117±0.007
*SBBR*	11	10 (90.90)	25 (7.12)	0.982±0.046	0.0213±0.012
CRT	20	17 (85.00)	37 (10.54)	0.984±0.021	0.0211±0.012
KZAZ	27	23 (85.20)	35 (9.97)	0.986±0.015	0.0157±0.009
KYR	52	38 (73.10)	61 (17.38)	0.988±0.006	0.0210±0.011
KKUR	51	43 (84.30)	70 (19.95)	0.988±0.008	0.0219±0.011
UZB	60	49 (81.67)	65 (18.51)	0.991±0.005	0.0215±0.011
*INMG*	16	13 (81.25)	25 (7.12)	0.975±0.029	0.0214±0.011
ALT	22	20 (90.90)	37 (10.54)	0.991±0.017	0.0205±0.011
*YUAN*	15	14 (93.33)	20 (5.70)	0.991±0.028	0.0128±0.007
TURKM	20	17 (85.00)	37 (10.54)	0.979±0.025	0.021±0.011
IRAN	233	176 (75.54)	120 (34.19)	0.995±0.002	0.0215±0.011
MONG	138	100 (72.46)	96 (27.35)	0.995±0.002	0.0223±0.012
KAL	99	85 (85.85)	79 (22.50)	0.996±0.002	0.0245±0.013
KAZ	40	37 (92.50)	58 (16.52)	0.996±0.007	0.0207±0.011
TAJ	20	20 (100)	43 (12.25)	1.000±0.016	0.0234±0.013
*AMGBR* *	3	3 (100)	5 (1.42)	1.000±0.272	0.0106±0.009

N- sample size, K- number of different haplotypes, S- number of polymorphic sites, *Ĥ*- gene diversity, π- nucleotide diversity.

* present study.

Ancient populations: AMGBR- Mongolia Altai Bronze Age, present study; PAZMG1- Mongolia Altai, Pazyryk, present study; PAZMG2- Mongolia Altai, Pazyryk; EGOL - Mongolia, Egyin Gol; PAZRA- Rep. Altai, Pazyryk; BRNRA- Rep. Altai, Neolithic and Bronze Age; SBBR- Siberia, Bronze Age; SBIR- Siberia, Iron Age; KZBR- Kazakhstan, Bronze Age; KZIR- Kazakhstan, Iron Age; LAJ- Lajia; YUAN- Xinjiang; INMG- Inner Mongolia. Current populations: CRT- Crimean Tartars; TURK- Turks; KZAZ- Kurds Zazaki; KKUR- Kurds Kurmanji; IRAN- Iraqis; KGEO- Georgians Kurds; GEOR- Georgians; KYR- Kirgiz; UZB- Uzbeks; KAZ- Kazaks; TURKM- Turkmens; TAJ- Tajiks; MONG- Mongols; TUV- Tuvans; TUB- Tubalars; ALT- Altaians; BUR- Buriats; KAL- Kalmiks; SIB- Siberians.

Additional information concerning each population can be found in Table S2.

Ancient populations are displayed in italic.

### Sex Determination

For genetic sex determination, X and Y Amelogenin loci and the SRY gene (sex-determining region Y gene) were analyzed using primers and conditions described respectively by Beraud-Colomb et al. [24] and Santos et al. [25].

**Figure 3:**
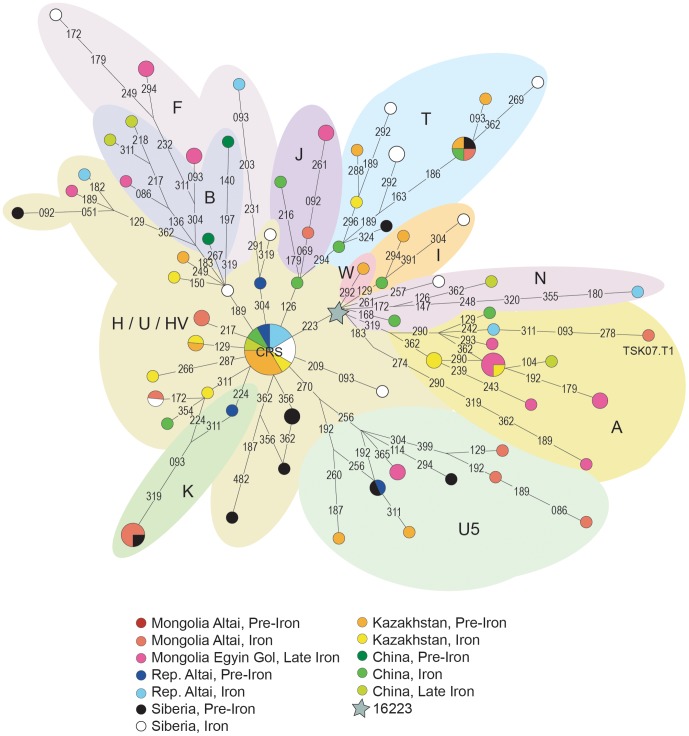
Median Joining Network of ancient N* haplogroup sequences. MtDNA sequences between positions 16051 and 16400, from ancient populations from the Mongolia (present study and [1], [2]), Russia [3], [4], [5], [6], Kazakhstan [7] and China [8], [9], [10] were used. Additional information concerning each population can be found in Table S2.

**Figure 4:**
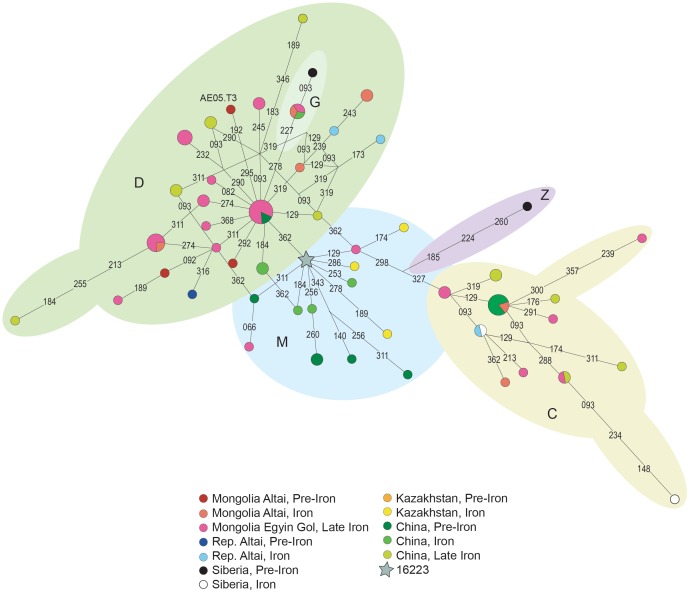
Median Joining Network of ancient M* haplogroup sequences. MtDNA sequences between positions 16051 and 16400, from ancient populations from the Mongolia (present study and [1], [2]), Russia [3], [4], [5], [6], Kazakhstan [7] and China [8], [9], [10] were used. Additional information concerning each population can be found in Table S2.

### Authentication of Results

Independent replication for four teeth and one bone were performed at the Institut de Biologia Evolutiva (CSIC-UPF) using the methodology previously described by Lalueza-Fox et al. [26]. Moreover, to authenticate the results, the recommended criteria concerning sterility, reproducibility, cloning, characterization of the investigators’ haplotype, coincidence of associated markers and diversity of the results were fulfilled. An integrative approach for human population studies was used, where the flexibility and the intelligent use of authentication criteria was applied [27], [28], [29].

**Figure 5:**
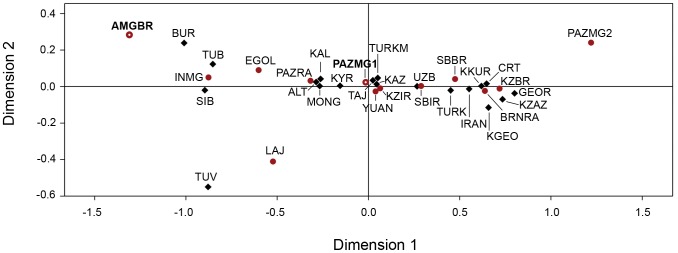
Multidimensional scaling representation of the Slatkin’s linearized F_ST_ pairwise genetic distance matrices between populations. Genetic distance based on HVRI variation of ancient and current Eurasian populations. Ancient populations (in red): AMGBR- Mongolia Altai Bronze Age, present study; PAZMG1- Mongolia Altai, Pazyryk, present study; PAZMG2- Mongolia Altai, Pazyryk; EGOL - Mongolia, Egyin Gol; PAZRA- Rep. Altai, Pazyryk; BRNRA- Rep. Altai, Neolithic and Bronze Age; SBBR- Siberia, Bronze Age; SBIR- Siberia, Iron Age; KZBR- Kazakhstan, Bronze Age; KZIR- Kazakhstan, Iron Age; LAJ- Lajia; YUAN- Xinjiang; INMG- Inner Mongolia. Current populations (in black): CRT- Crimean Tartars; TURK- Turks; KZAZ- Kurds Zazaki; KKUR- Kurds Kurmanji; IRAN- Iraqis; KGEO- Georgians Kurds; GEOR- Georgians; KYR- Kirgiz; UZB- Uzbeks; KAZ- Kazaks; TURKM- Turkmens; TAJ- Tajiks; MONG- Mongols; TUV- Tuvans; TUB- Tubalars; ALT- Altaians; BUR- Buriats; KAL- Kalmiks; SIB- Siberians. Additional information concerning each population can be found in Table S2.

### Data Analysis

Sequence raw data was analysed with Sequence Scanner v1.0 (Applied BioSystems) program and sequences were subsequently aligned with BioEdit software version 7.0.0 [30] in relation to the revised Cambridge Reference Sequence [31]. Samples were assigned to haplogroups using the combined information of HVRI and coding region variation following the phylogenetic classification updated by [32].

Haplogroups were clustered according to their geographic origin following [33], [34], [35]:

- West Eurasian haplogroups: R0: R0a’b, HV; N1; JT; UK; W and X.
- East Eurasian haplogroups: M: C, D, G, Z, M9, M10, M11, M13; A; B; F and N9a.
- South Asian haplogroups: M*, U2a-c, U9, R*, R1–R2, R5–R6, N1d.

For comparative purposes, mtDNA sequences between positions 16051 and 16400, from ancient [10], [11], [18], [19], [20], [36], [37], [38], [39], [40] and modern populations [3], [41], [42], [43], [44], [45], [46], [47], [48], [49], [50], [51], [52], [53], [54], [55], [56], [57] of Eastern and Central Asia, were collected (See Table S2, for population codes and details about period, sample size and references).

For each population, the number of different haplotypes (K), the number of polymorphic sites (S) [58], the gene diversity (Ĥ) [59] and the nucleotide diversity (π) [58], [59] were estimated using the software Arlequín ver. 3.11 [60].

Slatkin’s linearized FST pairwise genetic distance matrices between population [61] were calculated using the software Arlequin ver. 3.11. Multidimensional scaling.

(MDS) was used to represent genetic distances in a two-dimensional space using SPSS ver. 17.0 (SPSS Inc.).

Phylogenetic networks [62] among haplotypes were constructed using the program Network 4.610 (www.fluxus-engineering.com). Positions of HVRI were weighted according to their site-specific mutation rate following the weight scheme proposed by [63].

Spatial frequency distribution maps of East Eurasian lineages in Pre-Iron Age and Iron Age periods were obtained using Surfer version 8.05 (Golden Software).

## Results

### Authenticity of Results

Ancient DNA was retrieved and replicable unambiguous results were obtained for the 19 individuals analysed. The genetic sex determination was possible in fourteen of the nineteen individuals (Table 1). With exception of one individual (BTG06.T10B), morphological and genetic sex diagnoses were concordant. In individual BTG06.T13 genetic sex based on Amelogenin and SRY genes was contradictory, indicating a false negative amplification of Y-Chromosome Amelogenin.

Table 2 shows the mtDNA results for the HVRI sequencing and for the analysis of phylogenetically informative coding region polymorphisms. In all the samples, there is a concordance between the haplogroup inferred using SNPs typed along the coding region and the one based on HVRI haplotype. No coincidences between ancient and researchers’ sequences were found.

The cloning process was applied to the most informative HVRI regions of all individuals. The results further verify that the data of the sequences obtained represent the consensus in each individual as shown in Table S3. Moreover, the samples replicated at the Institut de Biologia Evolutiva yielded the same sequences found in the Universitat Autònoma de Barcelona, and proved the independent reproducibility of the results generated.

### Haplogroup Frequency and Sequence Diversity

The majority of the retrieved sequences (58%) fit into East Eurasian lineages; namely to haplogroups A, C, D and G. On the other hand, 42% of the individuals belong to West Eurasian mtDNA haplogroups (J, K, HV, U, and T haplogroups) (Table 2). Considering the chronology of burials, the three Bronze Age samples represent three different haplotypes all of which can be classified as haplogroup D. For Iron Age samples, the same proportion (50%) of East and West Eurasian lineages were found (Table 2).

Evidence of a perfect admixture between East Eurasian and West Eurasian lineages is also observed in other Iron Age populations from central Asia (Table 3 and Fig. 2B). On the other hand, in Neolithic and Bronze Age populations around 100% of the mtDNA lineages belong to East Eurasian or to Western Eurasian haplogroups, depending on the geographic location (Table 3 and Fig. 2A).

The number of mtDNA haplotypes, Nei gene diversity [59] and nucleotide diversity [58], [59] (based on HVRI sequences) for the studied populations and for populations selected for comparison are presented in table 4. Our Bronze Age samples of Mongolian Altai (AMGBR) display the highest value of gene diversity. However, this value must be interpreted with caution since only 3 individuals were analysed and the error associated to this estimation is the highest reported. Concerning our Iron Age population (PAZMG1), the value of diversity is in the range of values observed in other ancient and present day populations from the same geographical area. Regarding the nucleotidic diversity, Bronze Age samples show the lowest values whereas Iron Age samples show high values.

### Phylogeographic Analysis

Network analysis using ancient Asia populations (Fig. 3 and 4) displays the presence of both West Eurasian and East Eurasian haplogroups. A good separation of haplotypes belonging to the same haplogroup was achieved and the samples analysed in this study are correctly positioned in the phylogeny.

Seven of the sixteen different haplotypes found in this study (43.75%) are shared with other ancient populations used for comparison. Concerning these haplotypes, it is evidenced that K and T1 West Eurasian lineages detected in our Pazyryk sample were also found in Bronze Age samples from Siberia and Kazakhstan (Fig. 3).

For non-shared haplotypes, additional haplotype comparisons were performed including present-day data of Eurasian populations (data deposited in EMPOP and data from [64] and [65]); this additional analysis allows verification that these haplotypes were also present in at least one Eurasian population, with the exception of two haplotypes from individuals AE05.T3 and TSK07.T1. Thus, 87.5% of the haplotypes are shared with other ancient or current Eurasian populations.

The mtDNA haplotypes of AE05.T3 and TSK07.T1, derived respectively from other samples of the same haplogroup, by two and three additional mutations (respectively, Figure 4 and 3). All the additional mutations found in these samples are located in positions considered mutational hotspots [66] and, some of them, have been associated to post-mortem DNA damage [67]. Notwithstanding, all the clones of both samples display the mentioned mutations and sample TSK07.T1 was replicated in the palaeogenetics laboratory of the Institut de Biologia Evolutiva. Thus, although they have an unusual haplotype, the results obtained for these two samples appear to be authentic.

A broad analysis of shared haplotypes in pre-Iron Age (present study and [18], [20], [40]) and Iron Age populations (present study and [10], [11], [18], [19], [20], [36], [40]) from Central Asia and South Siberia demonstrate that only a small fraction 7 out of 72 (9.7%) of lineages are shared among populations. Three haplotypes (all with a West Eurasian origin) are shared between Iron Age and pre-Iron Age populations, whereas four are shared between Iron Age populations. Concerning non-shared haplotypes, 10 from 13 (77%) in pre-Iron Age populations, and 16 from 52 (31%) in Iron Age populations, represent West Eurasian lineages. Thus, it seems that the number of West Eurasian lineages does not increase in the Iron Age.

The Multidimensional Scaling (MDS) representation of the Slatkin genetic distance between pairs of ancient and modern populations [61] shows a separation of populations, across dimension 1, mainly based on the gradient of East and West Eurasian lineages (Fig. 5). Iron Age populations are together in the centre of coordinates showing an admixture of East Eurasian and West Eurasian lineages. Present day populations from Central Asia and Mongolia are grouped with ancient populations from the Iron Age.

With exception of the Pazyryk samples from Mongolia previously reported by [11], F_ST_ values for pairs of populations reveal that the Pazyryk populations seem to be genetically homogeneous. Notwithstanding, according to [11], these three samples belong to closely related individuals and cannot be considered representative of the population. Moreover, the Pazyryk groups (excluding [11]) did not present significant genetic differences with current Altaians. Although due to the small sample size of the Pazyryk groups these results must be interpreted with caution.

## Discussion

Central Asians exhibit high frequencies of East Eurasian mtDNA lineages, which are otherwise virtually absent in populations from the Indo-Gangetic region and westwards, along with a high prevalence of lineages of Western Eurasian origin [3], [33], [68], [69]. Also, high values of genetic diversity have been reported using different genetic systems. Two hypotheses have been put forward to explain this evidence: 1) Central Asians could represent an early incubator of Eurasian variation, or 2) their current genetic diversity could result from later admixture between West and East Eurasian populations. In the light of current knowledge, it seems that the second hypothesis is the most probable [3], [33] and although it is not possible to pinpoint all the process that generated the central Asian diversity, it is clear that the different gene pools that merged in their formation had already diverged on the outskirts of the Eurasian continent [3].

It is widely accepted that animal domestication (starting ∼5,000 YBP), particularly of the horse, gave the inhabitants of the Central Asian steppes the opportunity to expand geographically in different directions [12]. Historical records and archaeological data indicate that the early populations’ movements across the Eurasian steppe involved Indo-European-speaking people, being most probably in the Altai Mountains the eastern boundary. By the time of the 3rd century BC, Turkic-speaking peoples from the Altai region began to migrate westwards, replacing Indo-European languages in parts of Central Asia [12], [13], [14], [70]. Today, the Altai region is home to numerous Turkic-speaking ethnic groups, with a mixed mtDNA gene pool between West and East Eurasia [71]. However, the origin of this genetic admixture was prior to westward Turkic migrations and may be traced back to the Iron Age in Central Asia, or even earlier in South Siberia [40], [72]. With the present study, we have aimed to unravel the early contacts between European and Asian people in the Altai region by analyzing ancient mtDNA in human remains from Bronze and Iron Age burials of Mongolian Altai.

The high rate of success of the genetic analyses performed in the present study, evidenced by the amplification of both nuclear and mitochondrial DNA, suggests that the DNA of the samples is well preserved. In accordance with other authors [18], this is the expected result in samples that originated in cold and arid environmental conditions such as those found at most of the Altai sites. The agreement between morphological and genetic sex diagnoses, the concordant results obtained using coding and non-coding regions of mtDNA, the high haplotype diversity in sequence motifs, the concordant results between different extracts of the same sample, the clone results and the independent replication of some samples in another ancient DNA laboratory, all guarantee the authenticity of the obtained results [27], [28], [29]. Moreover, in terms of phylogenetic consistency, the observation of 16 different and phylogenetically plausible haplotypes among 19 ancient individuals and the coherence of the phylogenetic networks build further points towards the authenticity of the results [73].

Concerning Bronze Age samples from the Mongolian Altai mountains analyzed in the present study, 100% of the mtDNA lineages (3 different lineages from 2 archaeological sites) belong to East Eurasian haplogroups, an opposite profile to that detected in the West side of the Altai [18], [20]. On the other hand, in the Iron Age samples of Mongolian Altai, the same proportion (50%) of East and Western Eurasian lineages were found, evidencing a perfect admixture between East and Western Eurasian lineages as in other Iron Age populations from central Asia and Siberia [10], [18], [19], [20], [40]. Combined with the previous studies performed so far in the Altai region, our results suggest that the Altai represented a boundary to gene flow up to the beginning of the Iron Age and that during the Scythian period of the Altai (5th to 3rd century BC) there were demographic events in the region that led to a population admixture in both sides of the Altai.

Half of the shared haplotypes between ancient populations from Central Asia and South Siberia represent lineages present in both pre-Iron Age and Iron Age populations and all of these lineages have a west Eurasian origin. Moreover, considering both shared and non-shared haplotypes, it seems that the number of West Eurasian lineages does not increase in the Iron Age. These results allow us to hypothesise that the substrate of mtDNA lineages is already present in pre-Iron Age populations of the central Asia and that in the Iron Age (Scythian period) a population expansion lead to the admixture of pre-existing lineages. Thus, the admixture profile observed in the region during the Iron Age would not derive from a migratory movement from west to east, as has been hypothesised, but would represent a local population expansion in different directions. This population expansion, however, would be probably be a consequence of the introduction of new technology by the adoption of a new culture, supporting the idea of cultural transmission against the demic diffusion during Scythian period.

The Pazyryk groups analysed so far appear to be genetically homogeneous and they did not present significant genetic differences to current Altaians. These results suggest that roots of the current genetic diversity and admixture of the Altai region in Central Asia could be traced back to the Iron Age.

## Supporting Information

Table S1 Description of primers used and their references.
(DOCX)

Table S2 Ancient and current populations used in comparative analysis. References corresponds to reference number in the manuscript.
(DOC)

Table S3 Alignment of clone sequences.
(PDF)
