# Supplementary material for: Tracing the Origin of the East-West Population Admixture in the Altai Region (Central Asia)
Source: PLoS One. 2012 Nov 9;7(11):e48904. doi: 10.1371/journal.pone.0048904 (PMC3494716; doi:10.1371/journal.pone.0048904)
Supplement: Table S1 — Description of primers used and their references. (DOCX) [file pone.0048904.s001.docx]

Table S1. Description of primers used and their references

| **Region** | | **Sequence** | **Amplicon Length** | **References** |
| --- | --- | --- | --- | --- |
| **HVRI** | 16190 - 16420 | 5’-CCCCATGCTTACAAGCAAGT -3’ | 231 bp | [74] |
|  |  | 5’-TGATTTCACGGAGGATGGTG -3’ |  | [75] |
|  | 16190 - 16339 | 5'-CCCCATGCTTACAAGCAAGT -3' | 150 bp | [74] |
|  |  | 5'-GTGCTATGTACGGTAAATGG-3' |  | [76] |
|  | 16292 - 16420 | 5'- CACCCTTAACAGTACATAGTAC-3' | 129 bp | [74] |
|  |  | 5'- TGATTTCACGGAGGATGGTG -3' |  | [75] |
|  | 16030 - 16230 | 5'- CATGGGGAAGCAGATTTGGG-3' | 201 bp | [23] |
|  |  | 5'- GATAGTTGAGGGTTGATTGCTG-3' |  | [23] |
| **Coding Region** | 663 *Hae III* | 5’-TGAAAATGTTTAGACGGGCCTCACATC-3’ | 120 bp | [77] |
|  |  | 5’-TAGAGGGTGAACTCACTGGGAAC-3’ |  | [77] |
|  | 13259 *Hinc II* | 5'-AATCGTAGCCTTCTCCACTTCA-3' | 181 bp | [77] |
|  |  | 5'-TCCTATTTTTCGAATATCTTGTTC-3' |  | [78] |
|  | 5176 *Alu I* | 5’-TAGGATGAATAATAGCAGCTCTACCG -3’ | 182 bp | [79] |
|  |  | 5’-GGGTGGATGGAATTAAGGGTGT -3’ |  | [77] |
|  | 7025 *Alu I* | 5’-CCGTAGGTGGCCTGACTGGC-3’ | 124 bp | [74] |
|  |  | 5’-TGATGGCAAATACAGCTCCT-3’ |  | [74] |
|  | 9052 *Hae II* | 5'-ACGCCTAACCGCTAACATTAC-3' | 104 bp | [74] |
|  |  | 5'-AGATGATAAGTGTAGAGGGAAG-3' |  | [74] |
|  | 12308 *Hinf I* | 5'-CACAAGAACTGCTAACTCATGC-3' | 123 bp | [80] |
|  |  | 5'-ATTACTTTTATTTGGAGTTGCACCAA-3' |  | [80] |
|  | 15606 *Alu I* | 5'-CCCACATCAAGCCCGAATG-3' | 104 bp | [74] |
|  |  | 5'-GATGAGGATGGATAGTAATAGG-3' |  | [74] |
|  | 4830 *HaeII* | 5’-CTATCCTCTTCAACAATATACTCT-3’ | 161 bp | [81] |
|  |  | 5’- ATGTGAGAAGAAGCAGGC -3’ |  | [81] |
|  | 13704 *Bst 0I* | 5'-TCACCCTAACAGGTCAACC-3' | 118 bp | [74] |
|  |  | 5'-ATGAGAAATCCTGCGAATAG-3' |  | [74] |
|  | 4577 *Nla III* | 5’-CACTCATCACACAGCGCTAAGC-3’ | 122 bp | [80] |
|  |  | 5’-TGGCAGCTTCTGTGGAAC-3’ |  | [80] |
| **Amelogenin Gene** | | 5'- CCCTGGGCTCTGTAAAGAATAGTG- 3' | X: 106 bp  Y: 112 bp | [24] |
|  |  | 5'- ATCAGAGCTTAAACTGGGAAGCTG- 3' |  | [24] |
| **SRY Gene** | | 5’- ATAAGTATCGACCTCGTCGGAA -3’ | 93 bp | [25] |
|  |  | 5’- GCACTTCGCTGCAGAGTACCGA -3’ |  | [25] |

References 23, 24 and 25 corresponds to reference number in the manuscript

74. Montiel R, Malgosa A, Francalacci P (2001) Authenticating Ancient Human Mitochondrial DNA. Hum Biol 73: 689-713.

75. Vigilant L, Stoneking M, Harpending H, Hawkes K, Wilson AC (1991) African populations and the evolution of human mitochondrial DNA. Science 253: 1503-1507.

76. Díaz N (2010) Bahía de Alcúdia, Mallorca: Un crisol genético en el Mediterráneo. PhD thesis. Bellaterra: Universitat Autònoma de Barcelona. 233 p. <http://www.tesisenred.net/bitstream/handle/10803/3712/ndv1de1.pdf?sequence=1>

77. Handt O, Krings M, Ward RH, S. P (1996) The retrieval of ancient human DNA sequences. Am J Hum Genet 59: 368-376.

78. Ward RH, Frazier BL, Dew-Jager K, S. P (1991) Extensive mitochondrial diversity within a single Amerindian tribe. Proc Natl Acad Sci USA 88: 8720-8724.

79. Stone AC, Stoneking M (1998) mtDNA analysis of a prehistoric Oneota population: implications for the peopling of the New World. Am J Hum Genet 62: 1153-1170.

80. Izagirre N, Duran LM, De La Rua C (1998) Genética y arqueología : Análisis molecular de ADN procedente de restos esquelético. Munibe Ciencias naturales 50: 3-14.

81. Xie CZ, Li CX, Cui YQ, Zhang QC, Fu YQ, et al. (2007) Evidence of ancient DNA reveals the first European lineage in Iron Age Central China. Proc Biol Sci 274: 1597-1601.
