# Supplementary material for: Tracing the Origin of the East-West Population Admixture in the Altai Region (Central Asia)
Source: PLoS One. 2012 Nov 9;7(11):e48904. doi: 10.1371/journal.pone.0048904 (PMC3494716; doi:10.1371/journal.pone.0048904)
Supplement: Table S2 — Ancient and current populations used in comparative analysis. References corresponds to reference number in the manuscript. (DOC) [file pone.0048904.s002.doc]

Table S2. Ancient and current populations used in comparative analysis. References corresponds to reference number in the manuscript

| **Country** | **Population/ Period** | **Code** | **N** | **References** |
| --- | --- | --- | --- | --- |
| ***Ancient Populations*** | | | | |
| Mongolia, Altai | Bronze Age | AMGBR | 3 | This Study |
| Mongolia, Altai | Pazyryk, Iron Age 5th-3rd BC | PAZMG1 | 16 | This Study |
| Mongolia, Altai | Pazyryk, Iron Age 4th-3rd BC | PAZMG2 | 3 | [11] |
| Mongolia, Egyin Gol | Xiongnu, Iron Age 3rd BC-2nd AD | EGOL | 46 | [36] |
| Russia, Rep. Altai | Neolithic and Bronze Age | BRNRA | 4 | [20] |
| Russia, Rep. Altai | Pazyryk, Iron Age 6th-3rd BC | PAZRA | 10 | [10, 19, 20] |
| Russia, Siberia | Bronze Age 19th-9th BC | SBBR | 11 | [40] |
| Russia, Siberia | Iron Age 9th BC-5th AD | SBIR | 15 | [40] |
| Kazakhstan | Bronze Age 14th-10th BC | KZBR | 13 | [18] |
| Kazakhstan | Iron Age 8th BC -1st BC | KZIR | 13 | [18] |
| China, Lajia | Qijia, Late Neolithic 4000 BP | LAJ | 14 | [38] |
| China, Xinjiang | Yuansha site, Iron Age 2135 BP | YUAN | 15 | [39] |
| China, Inner Mongolia | Tuoba Xianbei, 4th-5th AD | INMG | 16 | [37] |
| **Current Populations** | | | | |
| Ukraine, Crimea | Crimean Tartars | CRT | 20 | [3] |
| Turkey | Turks | TURK | 74 | [47] |
| Iran | Kurds Zazaki | KZAZ | 27 | [49] |
| Iran | Kurds Kurmanji | KKUR | 51 | [49] |
| Iran | Iraqis | IRAN | 233 | [3, 51, 56] |
| Georgia | Georgians Kurds | KGEO | 29 | [44] |
| Georgia | Georgians | GEOR | 45 | [44] |
| Kirghizstan | Kirgiz | KYR | 52 | [3] |
| Uzbekistan | Uzbeks | UZB | 60 | [3] |
| Kazakhstan | Kazaks | KAZ | 40 | [3] |
| Turkmenistan | Turkmens | TURKM | 20 | [3] |
| Tajikistan | Tajiks | TAJ | 20 | [3] |
| Mongolia | Mongols | MONG | 138 | [45, 55] |
| South Russia | Tuvans | TUV | 63 | [42, 50, 52, 57] |
| South Russia | Tubalars | TUB | 11 | [50, 52, 57] |
| South Russia | Altaians | ALT | 22 | [41, 53] |
| South Russia | Buriats | BUR | 33 | [42, 50, 53, 57] |
| East Russia | Kalmiks | KAL | 99 | [48] |
| Russia | Siberians | SIB | 515 | [42, 43, 46, 50, 52, 54,57] |
