# Supplementary material for: Tracing the Origin of the East-West Population Admixture in the Altai Region (Central Asia)
Source: PLoS One. 2012 Nov 9;7(11):e48904. doi: 10.1371/journal.pone.0048904 (PMC3494716; doi:10.1371/journal.pone.0048904)
Supplement: Table S3 — Alignment of clone sequences. (PDF) [file pone.0048904.s003.pdf]

Table S3.: Alignment of clone sequences (* Samples replicated in the Institut de Biologia Evolutiva)

AE05.T2

```
      11111111111111111111111111111111111111111111111111111111111111111111111111111111111111111111111111111111111111111111111111111111111111111111111111111111111111111111111111111111111111111111111111111111111111111111111111111111111111
      66666666666666666666666666666666666666666666666666666666666666666666666666666666666666666666666666666666666666666666666666666666666666666666666666666666666666666666666666666666666666666666666666666666666666666666666666666666666666
      11111222222222222222222222222222222222222222222222222222222222222222222222222222222222222222222222222222233333333333333333333333333333333333333333333333333333333333333333333333333333333333333333333333333333334444444444444444444444444444444
      99999000000000011111111112222222222333333333344444444445555555555666666666677777777778888888888999999999900000000001111111111222222222233333333334444444444555555555566666666667777777777888888888899999999990000000000111111111122222222223
      56789012345678901234567890123456789012345678901234567890123456789012345678901234567890123456789012345678901234567890123456789012345678901234567890123456789012345678901234567890123456789012345678901234567890123456789012345678901234567890
      TGCTTACAAGCAAGTACAGCAATCAACCCTCAACTATCACACATCAACTGCAACTCCAAAGCCACCCCTCACCCACTAGGATACCAACAAACCTACCCACCCTTAACAGTACATAGTACATAAAGCCATTTACCGTACATAGCACATTACAGTCAAATCCCTTCTCGTCCCCATGGATGACCCCCCTCAGATAGGGGTCCCTTGACCACCATCCTCCGTGAAATCAATATCCCGCA
Cl1   ............................T.....................................................................T.....................................................................C..........................................................
Cl2   .....................G..G...T..........................................................................................................................................C.T........................................................
Cl3   ............................T.....................................................................T.....................................................................C..........................................................
Cl4   ............................T.....................G............G.......................................................................................................C..........................................................
Cl5   ............................T.....................................................................T.....................................................................C..........................................................
Cl6   ............................T.....................................................................T.....................................................................C..........................................................
Cl7   ............................T..................................G.......................................................................................................C.T........................................................
Cl8   ............................T....................................................................................................G.....................................C..........................................................
Cl9   ............................T.....................................................................T.....................................................................C..........................................................
Cl10  ............................T..........................................................................................................................................C.T........................................................
Cl11  ............................T.....................................................................T.....................................................................C..........................................................
Cl12  ............................T.....................................................................T.....................................................................C..........................................................
Cl13  ............................T.....................................................................T.....................................................................C..........................................................
Cl14  ............................T.....................................................................T.....................................................................C..........................................................
```

AE05.T3

```
      11111111111111111111111111111111111111111111111111111111111111111111111111111111111111111111111111111111111111111111111111111111111111111111111111111111111111111111111111111111111111111111111111111111111111111111111111111111111111
      66666666666666666666666666666666666666666666666666666666666666666666666666666666666666666666666666666666666666666666666666666666666666666666666666666666666666666666666666666666666666666666666666666666666666666666666666666666666666
      11111222222222222222222222222222222222222222222222222222222222222222222222222222222222222222222222222222233333333333333333333333333333333333333333333333333333333333333333333333333333333333333333333333333333334444444444444444444444444444444
      99999000000000011111111112222222222333333333344444444445555555555666666666677777777778888888888999999999900000000001111111111222222222233333333334444444444555555555566666666667777777777888888888899999999990000000000111111111122222222223
      56789012345678901234567890123456789012345678901234567890123456789012345678901234567890123456789012345678901234567890123456789012345678901234567890123456789012345678901234567890123456789012345678901234567890123456789012345678901234567890
      TGCTTACAAGCAAGTACAGCAATCAACCCTCAACTATCACACATCAACTGCAACTCCAAAGCCACCCCTCACCCACTAGGATACCAACAAACCTACCCACCCTTAACAGTACATAGTACATAAAGCCATTTACCGTACATAGCACATTACAGTCAAATCCCTTCTCGTCCCCATGGATGACCCCCCTCAGATAGGGGTCCCTTGACCACCATCCTCCGTGAAATCAATATCCCGCA
Cl1   ............................T........................................................................T..................................................................C.........................................................
Cl2   ........................G...T..................................G....................................T............................G.....................................C.........................................................
Cl3   ............................T........................................................................T..................................................................C.........................................................
Cl4   ........N...............G...T.....................GGG..........G....................................T........G.........................................................C.........................................................
Cl5   ............................T........................................................................T..................................................................C.........................................................
Cl6   ............................T........................................................................T..................................................................C.........................................................
Cl7   ............................T........................................................................T..................................................................C.........................................................
Cl8   ............................T........................................................................T..................................................................C.........................................................
Cl9   ............................T........................................................................T..................................................................C.........................................................
Cl10  ............................T........................................................................T..................................................................C.........................................................
```

TSA07.T4*

```
      111111111111111111111111111111111111111111111111111111111111111111111111111111111111111111111111111111111111111111111111111111111111111111111111111111111111111111111111111111111111111111111111111111111111111111111111111111111111111111111111111111111111111111111111111111111111111111111111111111111111111111111111111111111111111111111111111111111111111111111111111111111111
      666666666666666666666666666666666666666666666666666666666666666666666666666666666666666666666666666666666666666666666666666666666666666666666666666666666666666666666666666666666666666666666666666666666666666666666666666666666666666666666666666666666666666666666666666666666666666666666666666666666666666666666666666666666666666666666666666666666666666666666666666666666666
      000000000000000000000000000000000000000000000000000000000000000000000000000001111111111111111111111111111111111111111111111111111111111111111111111111111111111111111111111111111111111122222222222222222222222222222222222222222222222222222222222222222222222222222222222222222222222222222222333333333333333333333333333333333333333333333333333333333333333333333333333333333333333333333333334
      222222233333333334444444444555555555566666666667777777777888888888899999999990000000000111111111122222222223333333333444444444455555555556666666666777777777788888888889999999999000000000011111111112222222222333333333344444444445555555555666666666677777777778888888888999999999900000000001111111111222222222233333333334444444444555555555566666666667777777777888888888899999999990
      345678901234567890123456789012345678901234567890123456789012345678901234567890123456789012345678901234567890123456789012345678901234567890123456789012345678901234567890123456789012345678901234567890123456789012345678901234567890123456789012345678901234567890123456789012345678901234567890123456789012345678901234567890123456789012345678901234567890123456789012345678901234567890
      GTTCTTTCATGGGGAAGCAGATTTGGGTACCACCCAAGTATTGACTCACCCATCAACAACCGCTATGTATTTCGTACATTACTGCCAGCCACCATGAATATTGTACGGTACCATAAATACTTGACCACCTGTAGTACATAAAAACCCAATCCACATCAAAACCCCCTCCCCATGCTTACAAGCAAGTACAGCAATCAACCCTCAACTATCACACATCAACTGCAACTCCAAAGCCACCCCTCACCCACTAGGATACCAACAAACCTACCCACCCTTAACAGTACATAGTACATAAAGCCATTTACCGTACATAGCACATTACAGTCAAATCCCTTCTCGTCCCCATGGATGACCCCCCTCAGATAGGGGTCCCTTGAC
Cl1   .....................................................................C......................................................................................................................
Cl2   .....................................................................C......................................................................................................................
Cl3   .....................................................................C...............T......................................................................................................
Cl4   .....................................................................C......................................................................................................................
Cl5   .....................................................................C......................................................................................................................
Cl6   .....................................................................C......................................................................................................................
Cl7   .....................................................................C......................................................................................................................
Cl8   .....................................................................C......................................................................................................................
Cl9   ...............................................................C............................................................................................................................
Cl10  .....................................................................C......................................................................................................................
Cl1                                                                                                                                                                           ....................................T.......................................................................................C..................................................C...............
Cl2                                                                                                                                                                           ....................................T.......................................................................................C..................................................C...............
Cl3                                                                                                                                                                           ....................................T.......................................................................................C..................................................C............T.T
Cl4                                                                                                                                                                           ....................................T.......................................................................................C..................................................C...............
Cl5                                                                                                                                                                           ....................................T........................................T..............................................C..................................................C...............
Cl6                                                                                                                                                                           ....................................T.......................................................................................C..................................................C...............
Cl7                                                                                                                                                                           ....................................T.......................................................................................C..................................................C...............
Cl8                                                                                                                                                                           ....................................T..................................................A....................................C..................................................C...............
Cl9                                                                                                                                                                           ....................................T..................................................................................T....C..................................................C...............
Cl10                                                                                                                                                                          ....................................T.......................................................................................C..................................................C...............
Cl11                                                                                                                                                                          ....................................T.......................................................................................C..................................................C...............
Cl12                                                                                                                                                                          ....................................T.......................................................................................C..................................................C...............
Cl13                                                                                                                                                                          ....................................T.......................................................................................C..................................................C...............
Cl14                                                                                                                                                                          ....................................T.......................................................................................C..................................................C...............
```

BTG05.T1

```
        11111111111111111111111111111111111111111111111111111111111111111111111111111111111111111111111111111111111111111111111111111111111111111111111111111111111111111111111111111111111111111111111111111111111111111111111111111111111111111111
        66666666666666666666666666666666666666666666666666666666666666666666666666666666666666666666666666666666666666666666666666666666666666666666666666666666666666666666666666666666666666666666666666666666666666666666666666666666666666666666
        11111222222222222222222222222222222222222222222222222222222222222222222222222222222222222222222222222222333333333333333333333333333333333333333333333333333333333333333333333333333333333333333333333333333333334444444444444444444444444444444
        99999000000000011111111112222222222333333333344444444445555555555666666666677777777778888888888999999999900000000001111111111222222222233333333334444444444555555555566666666667777777777888888888899999999990000000000111111111122222222223
        56789012345678901234567890123456789012345678901234567890123456789012345678901234567890123456789012345678901234567890123456789012345678901234567890123456789012345678901234567890123456789012345678901234567890123456789012345678901234567890
        TGCTTACAAGCAAGTACAGCAATCAACCCTCAACTATCACACATCAACTGCAACTCCAAAGCCACCCCTCACCCACTAGGATACCAACAAACCTACCCACCCTTAACAGTACATAGTACATAAAGCCATTTACCGTACATAGCACATTACAGTCAAATCCCTTCTCGTCCCCATGGATGACCCCCCTCAGATAGGGGTCCCTTGACCACCATCCTCCGTGAAATCAATATCCCGCA
C11     ............................T...............................................................................................A......................
C12     ............................T...................C..............................................T............................A......................
C13     ............................T...............................................................................................A......................
C14     ............................T...............................................................................................A......................
C15     ............................T...............................................................................................A......................
C16     ............................T........C......................................................................................A......................
C17     ............................T...............................................................................................A......................
C18     ............................T...................................T....T......................................................A......................
C19     ............................T...............................................................................................A......................
C110    ............................T...............................................................................................A......................
C11                                                                                                              ...........................A..........................................C...........................................................
C12                                                                                                              ...........................A..........................................C...........................................................
C13                                                                                                              ...........................A..........................................C.........................A.................................
C14                                                                                                              ...........................A..........................................C...........................................................
C15                                                                                                              ...........................A..........................................C.......A...................................................
C16                                                                                                              ...........................A..........................................C...........................................................
C17                                                                                                              ...........................A..........................................C...........................................................
C18                                                                                                              ...........................A..........................................C...........................................................
```

BTG05.T2

```
        11111111111111111111111111111111111111111111111111111111111111111111111111111111111111111111111111111111111111111111111111111111111111111111111111111111111111111111111111111111111111111111111111111111111111111111111111111111111111111111
        66666666666666666666666666666666666666666666666666666666666666666666666666666666666666666666666666666666666666666666666666666666666666666666666666666666666666666666666666666666666666666666666666666666666666666666666666666666666666666666
        11111222222222222222222222222222222222222222222222222222222222222222222222222222222222222222222222222222333333333333333333333333333333333333333333333333333333333333333333333333333333333333333333333333333333334444444444444444444444444444444
        99999000000000011111111112222222222333333333344444444445555555555666666666677777777778888888888999999999900000000001111111111222222222233333333334444444444555555555566666666667777777777888888888899999999990000000000111111111122222222223
        56789012345678901234567890123456789012345678901234567890123456789012345678901234567890123456789012345678901234567890123456789012345678901234567890123456789012345678901234567890123456789012345678901234567890123456789012345678901234567890
        TGCTTACAAGCAAGTACAGCAATCAACCCTCAACTATCACACATCAACTGCAACTCCAAAGCCACCCCTCACCCACTAGGATACCAACAAACCTACCCACCCTTAACAGTACATAGTACATAAAGCCATTTACCGTACATAGCACATTACAGTCAAATCCCTTCTCGTCCCCATGGATGACCCCCCTCAGATAGGGGTCCCTTGACCACCATCCTCCGTGAAATCAATATCCCGCA
C11     .............................C......................................................................................C.......A.......................
C12     .............................C......................................................................................C.......A.......................
C13     .............................C.......................................N.........A...T................................C.......A.......................
C14     .............................C......................................................................................C.......A.......................
C15     .............................C......................................................................................C.......A.......................
C16     .............................C......................................................................................C.......A.......................
C17     .............................C......................................................................................C.......A.......................
C18     .............................C......................................................................................C.......A.......................
C11                                                                                                              ...................C.......A......................................................................................................
C12                                                                                                              ...................C.......A......................................................................................................
C13                                                                                                              ...................C.......A......................................................................................................
C14                                                                                                              ...................C.......A......................................................................................................
C15                                                                                                              ...................C.......A......................................................................................................
C16                                                                                                              ...................C.......A......................................................................................................
C17                                                                                                              ...................C.......A......................................................................................................
C18                                                                                                              ...................C.......A......................................................................................................
```

BTG05.T8.1

```
        11111111111111111111111111111111111111111111111111111111111111111111111111111111111111111111111111111111111111111111111111111111111111111111111111111111111111111111111111111111111111111111111111111111111111111111111111111111111111111111
        66666666666666666666666666666666666666666666666666666666666666666666666666666666666666666666666666666666666666666666666666666666666666666666666666666666666666666666666666666666666666666666666666666666666666666666666666666666666666666666
        11111222222222222222222222222222222222222222222222222222222222222222222222222222222222222222222222222222333333333333333333333333333333333333333333333333333333333333333333333333333333333333333333333333333333334444444444444444444444444444444
        99999000000000011111111112222222222333333333344444444445555555555666666666677777777778888888888999999999900000000001111111111222222222233333333334444444444555555555566666666667777777777888888888899999999990000000000111111111122222222223
        56789012345678901234567890123456789012345678901234567890123456789012345678901234567890123456789012345678901234567890123456789012345678901234567890123456789012345678901234567890123456789012345678901234567890123456789012345678901234567890
        TGCTTACAAGCAAGTACAGCAATCAACCCTCAACTATCACACATCAACTGCAACTCCAAAGCCACCCCTCACCCACTAGGATACCAACAAACCTACCCACCCTTAACAGTACATAGTACATAAAGCCATTTACCGTACATAGCACATTACAGTCAAATCCCTTCTCGTCCCCATGGATGACCCCCCTCAGATAGGGGTCCCTTGACCACCATCCTCCGTGAAATCAATATCCCGCA
C11     .............................................................T.............T.................................C..............................................................................................G......................
C12     .............................................................T.............T.................................C..............................................................................................G......................
C13     .............................................................T.............T.................................C..............................................................................................G......................
C14     .............................................................T.............T.................................C..............................................................................................G......................
C15     .............................................................T.............T.................................C..............................................................................................G......................
C16     .............................................................T.............T.................................C..............................................................................................G......................
C17     ..............................................G..............T.............T.................................C..............................................................................................G......................
C18     .............................................................T.............T.................................C..............................................................................................G......................
C19     .............................................................T.............T.................................C..............................................................................................G......................
C110    .............................................................T.............T.................................C..............................................................................................G......................
```

BTG05.T8.2

```
            11111111111111111111111111111111111111111111111111111111111111111111111111111111111111111111111111111111111111111111111111111111111111111111111111111111111111111111111111111111111111111111111111111111111111111111111111111111111111111111
            66666666666666666666666666666666666666666666666666666666666666666666666666666666666666666666666666666666666666666666666666666666666666666666666666666666666666666666666666666666666666666666666666666666666666666666666666666666666666666666
            11111222222222222222222222222222222222222222222222222222222222222222222222222222222222222222222222222222333333333333333333333333333333333333333333333333333333333333333333333333333333333333333333333333333333334444444444444444444444444444444
            99999000000000011111111112222222222333333333344444444445555555555666666666677777777778888888888999999999900000000001111111111222222222233333333334444444444555555555566666666667777777777888888888899999999990000000000111111111122222222223
            56789012345678901234567890123456789012345678901234567890123456789012345678901234567890123456789012345678901234567890123456789012345678901234567890123456789012345678901234567890123456789012345678901234567890123456789012345678901234567890
            TGCTTACAAGCAAGTACAGCAATCAACCCTCAACTATCACACATCAACTGCAACTCCAAAGCCACCCCTCACCCACTAGGATACCAACAAACCTACCCACCCTTAACAGTACATAGTACATAAAGCCATTTACCGTACATAGCACATTACAGTCAAATCCCTTCTCGTCCCCATGGATGACCCCCCTCAGATAGGGGTCCCTTGACCACCATCCTCCGTGAAATCAATATCCCGCA
Cl1         ............................T..........................................................................C............................T.............................................................................................
Cl2         ............................T..........................................................................C..............T.............T.............................................................................................
Cl3         ............................T..........................................................................C............................T.............................................................................................
Cl4         ............................T..........................................................................C............................T.............................................................................................
Cl5         ............................T..........................................................................C..............T.............T.............................................................................................
Cl6         ............................T..........................................................................C............................T.............................................................................................
```

BTG05.T8.3*

```
            11111111111111111111111111111111111111111111111111111111111111111111111111111111111111111111111111111111111111111111111111111111111111111111111111111111111111111111111111111111111111111111111111111111111111111111111111111111111111111111
            66666666666666666666666666666666666666666666666666666666666666666666666666666666666666666666666666666666666666666666666666666666666666666666666666666666666666666666666666666666666666666666666666666666666666666666666666666666666666666666
            11111222222222222222222222222222222222222222222222222222222222222222222222222222222222222222222222222222333333333333333333333333333333333333333333333333333333333333333333333333333333333333333333333333333333334444444444444444444444444444444
            99999000000000011111111112222222222333333333344444444445555555555666666666677777777778888888888999999999900000000001111111111222222222233333333334444444444555555555566666666667777777777888888888899999999990000000000111111111122222222223
            56789012345678901234567890123456789012345678901234567890123456789012345678901234567890123456789012345678901234567890123456789012345678901234567890123456789012345678901234567890123456789012345678901234567890123456789012345678901234567890
            TGCTTACAAGCAAGTACAGCAATCAACCCTCAACTATCACACATCAACTGCAACTCCAAAGCCACCCCTCACCCACTAGGATACCAACAAACCTACCCACCCTTAACAGTACATAGTACATAAAGCCATTTACCGTACATAGCACATTACAGTCAAATCCCTTCTCGTCCCCATGGATGACCCCCCTCAGATAGGGGTCCCTTGACCACCATCCTCCGTGAAATCAATATCCCGCA
Cl1         ............................T...............T...C...........................................................................A..........................................C.......A.............................
Cl2         ............................T...............T...C..................T........................................................A..........................................C.......A.............................
Cl3         ............................T...............T...C..................T.T.T....................................................A..........................................C.....................................
Cl4         ............................T...............T...C...........................................................................A..........................................C.....................................
Cl5         ............................T...............T...C...........................................................................A..........................................C........G............................
Cl6         ............................T...............T...C...........................................................................A..........................................C.......A.............................
```

BTG06.T3

```
            11111111111111111111111111111111111111111111111111111111111111111111111111111111111111111111111111111111111111111111111111111111111111111111111111111111111111111111111111111111111111111111111111111111111111111111111111111111111111111111
            66666666666666666666666666666666666666666666666666666666666666666666666666666666666666666666666666666666666666666666666666666666666666666666666666666666666666666666666666666666666666666666666666666666666666666666666666666666666666666666
            00000000000000000000000000000000000000000000000000000000000000000000000000000111111111111111111111111111111111111111111111111111111111111111111111111111111111111111111111111111111111111111111111111111111111111111111111111111111111111111111
            22222223333333333444444444455555555556666666666777777777788888888889999999999000000000011111111112222222222333333333344444444445555555555666666666677777777778888888888999999999900000000001111111111222222222233333333334444444444555555555
            34567890123456789012345678901234567890123456789012345678901234567890123456789012345678901234567890123456789012345678901234567890123456789012345678901234567890123456789012345678901234567890123456789012345678901234567890123456789012345678
            GTTCTTTCATGGGGAAGCAGATTTGGGTACCACCCAAGTATTGACTCACCCATCAACAACCGCTATGTATTTCGTACATTACTGCCAGCCACCATGAATATTGTACGGTACCATAAATACTTGACCACCTGTAGTACATAAAAACCCAATCCACATCAAAACCCCCTCCCCATGCTTACAAGCAAGTACAGCAATCAACCCTCAACTATCATAGCACATTACAGTCAAATCCCTTC
Cl1              .........................................T........................................................C............................................................................................................
Cl2              .........................................T........................................................C............................................................................................................
Cl3              .........................................T........................................................C............................................................................................................
Cl4              .........................................T........................................................C............................................................................................................
Cl5              ........................T................T.........T..T..T........................................C............................................................................................................
Cl6              .........................................T...............T........................................C............................................................................................................
```

BTG06.T8*

```
            111111111111111111111111111111111111111111111111111111111111111111111111111111111111111111111111111111111111111111111111111111111111111111111111111111111111111111111111111111111111111111111111111111111111111111111111111111111111111111111111111111111111111111111111111111111111111111111111111111111111111111111111111111111111111111111111111111111111111111111111111111111111
            666666666666666666666666666666666666666666666666666666666666666666666666666666666666666666666666666666666666666666666666666666666666666666666666666666666666666666666666666666666666666666666666666666666666666666666666666666666666666666666666666666666666666666666666666666666666666666666666666666666666666666666666666666666666666666666666666666666666666666666666666666666666
            000000000000000000000000000000000000000000000000000000000000000000000000000001111111111111111111111111111111111111111111111111111111111111111111111111111111111111111111111111111111111111111111222222222222222222222222222222222222222222222222222222222222222222222222222222222222222222222222222222333333333333333333333333333333333333333333333333333333333333333333333333333333333333333333333333334
            222222233333333334444444444555555555566666666667777777777888888888899999999990000000000111111111122222222223333333333444444444455555555556666666666777777777788888888889999999999000000000011111111112222222222333333333344444444445555555555666666666677777777778888888888999999999900000000001111111111222222222233333333334444444444555555555566666666667777777777888888888899999999990
            345678901234567890123456789012345678901234567890123456789012345678901234567890123456789012345678901234567890123456789012345678901234567890123456789012345678901234567890123456789012345678901234567890123456789012345678901234567890123456789012345678901234567890123456789012345678901234567890123456789012345678901234567890123456789012345678901234567890123456789012345678901234567890
            GTTCTTTCATGGGGAAGCAGATTTGGGTACCACCCAAGTATTGACTCACCCATCAACAACCGCTATGTATTTCGTACATTACTGCCAGCCACCATGAATATTGTACGGTACCATAAATACTTGACCACCTGTAGTACATAAAAACCCAATCCACATCAAAACCCCCTCCCCATGCTTACAAGCAAGTACAGCAATCAACCCTCAACTATCACACATCAACTGCAACTCCAAAGCCACCCCTCACCCACTAGGATACCAACAAACCTACCCACCCTTAACAGTACATAGTACATAAAGCCATTTACCGTACATAGCACATTACAGTCAAATCCCTTCTCGTCCCCATGGATGACCCCCCTCAGATAGGGGTCCCTTGAC
Cl1         ........................................................................T..............................C....................................................................................
Cl2         ............................................................................................................................................................................................
Cl3         ..................................T.....................................................................................................................................................
Cl4         ............................................................................................................................................................................................
Cl5         ...............................G............................T...........................................................................................................................
Cl6         ............................................................................................................................................................................................
Cl7         ............................................................................................................................................................T...........T...................
Cl8         .................................................T...T......T.T.............T...........T...................................................................................................
Cl9         ............................................................................................................................................................................................
Cl10        .................................................T...T......T.T.............T...........T...................................................................................................
Cl11        .............................................................A..............................................................................................................................
Cl12        ............................................................................................................................................................................................
Cl13        ..................................T.....................................................................................................................................................
Cl14        ............................................................................................................................................................................................
Cl1                                                                                                                                                                                 ....................................T...............T...C...........................................................................A..........................................C...............
Cl2                                                                                                                                                                                 ....................................T...............T...C..................T........................................................A..........................................C...............
Cl3                                                                                                                                                                                 ....................................T...............T...C..................T.T.T....................................................A..........................................C...............
Cl4                                                                                                                                                                                 ....................................T...............T...C...........................................................................A..........................................C...............
Cl5                                                                                                                                                                                 ....................................T...............T...C...........................................................................A..........................................C...............
Cl6                                                                                                                                                                                 ....................................T...............T...C...........................................................................A..........................................C...............
Cl7                                                                                                                                                                                 ....................................................T...C...........................................................................A...................................T......C...............
Cl8                                                                                                                                                                                 ....................................T.....C.........T...C...........................................................................A..........................................C...............
Cl9                                                                                                                                                                                 ....................................T...............T...C...........................................................................A...................................T......C...............
```

BTG06.T10A

```
          11111111111111111111111111111111111111111111111111111111111111111111111111111111111111111111111111111111111111111111111111111111111111111111111111111111111111111111111111111111111111111111111111111111111111111111111111111111111111111111111
          66666666666666666666666666666666666666666666666666666666666666666666666666666666666666666666666666666666666666666666666666666666666666666666666666666666666666666666666666666666666666666666666666666666666666666666666666666666666666666666
          00000000000000000000000000000000000000000000000000000000000000000000000000000111111111111111111111111111111111111111111111111111111111111111111111111111111111111111111111111111122222222222222222222222222222222222222222222222222222222222
          22222223333333333444444444455555555556666666666777777777788888888889999999999000000000011111111112222222222333333333344444444445555555555666666666677777777778888888888999999999900000000001111111111222222222233333333334444444444555555555
          34567890123456789012345678901234567890123456789012345678901234567890123456789012345678901234567890123456789012345678901234567890123456789012345678901234567890123456789012345678901234567890123456789012345678901234567890123456789012345678
          GTTCTTTCATGGGGAAGCAGATTTGGGTACCACCCAAGTATTGACTCACCCATCAACAACCGCTATGTATTTCGTACATTACTGCCAGCCACCATGAATATTGTACGGTACCATAAATACTTGACCACCTGTAGTACATAAAAACCCAATCCACATCAAAACCCCCTCCCCATGCTTACAAGCAAGTACAGCAATCAACCCTCAACTATCATAGCACATTACAGTCAAATCCCTTC
C11            ................................................T...............................................................................................C..............................................................
C12            ................................................................................................................................................C..............................................................
C13            ................................................................................................................................................C..............................................................
C14            ................................................................................................................................................C..............................................................
C15            ................................................................................................................................................C..............................................................
C16            ................................................................................................................................................C..............................................................
C17            ....................................................G...........................................................................................C..............................................................
C18            ................................................................................................................................................C..............................................................
C19            ................................................................................................................................................C..............................................................
C110           ................................................................................................................................................C..............................................................
C111           ................................................................................................................................................C..............................................................
C112           ................................................................................................................................................C..............................................................
C113           ................................................................................................................................................C..............................................................
C114           ................................................................................................................................................C..............................................................
C115           ................................................................................................................................................C..............................................................
```

BTG06.T10B

```
          11111111111111111111111111111111111111111111111111111111111111111111111111111111111111111111111111111111111111111111111111111111111111111111111111111111111111111111111111111111111111111111111111111111111111111111111111111111111111111111111
          66666666666666666666666666666666666666666666666666666666666666666666666666666666666666666666666666666666666666666666666666666666666666666666666666666666666666666666666666666666666666666666666666666666666666666666666666666666666666666666
          11111222222222222222222222222222222222222222222222222222222222222222222222222222222222222222222222222222233333333333333333333333333333333333333333333333333333333333333333333333333333333333333333333333333333334444444444444444444444444444444
          99999000000000011111111112222222222333333333344444444445555555555666666666677777777778888888888999999999900000000001111111111222222222233333333334444444444555555555566666666667777777777888888888899999999990000000000111111111122222222223
          56789012345678901234567890123456789012345678901234567890123456789012345678901234567890123456789012345678901234567890123456789012345678901234567890123456789012345678901234567890123456789012345678901234567890123456789012345678901234567890
          TGCTTACAAGCAAGTACAGCAATCAACCCTCAACTATCACACATCAACTGCAACTCCAAAGCCACCCCTCACCCACTAGGATACCAACAAACCTACCCACCCTTAACAGTACATAGTACATAAAGCCATTTACCGTACATAGCACATTACAGTCAAATCCCTTCTCGTCCCCATGGATGACCCCCCTCAGATAGGGGTCCCTTGACCACCATCCTCCGTGAAATCAATATCCCGCA
C11       ............................T..................................................A....................................C..................................................C..........................................................
C12       ............................T..................................................A....................................C..................................................C..........................................................
C13       ............................T...................G..............................A....................................C..................................................C..........................................................
C14       ............................T..................................................A....................................C..................................................C..........................................................
C15       ............................T..................................................A....................................C..................................................C..........................................................
C16       ............................T...................G..............................A....................................C..................................................C..........................................................
C17       .....................G......T...................G..............................A....................................C..................................................C..........................................................
C18       ............................T...................G..............................A....................................C..................................................C..........................................................
C19       ............................T..................................................A....................................C..................................................C..........................................................
C110      ............................T..................................................A....................................C..................................................C..........................................................
C111      ............................T..................................................A....................................C..................................................C..........................................................
C112      ............................T..................................................A....................................C..................................................C..........................................................
C113      ............................T..................................................A....................................C..................................................C..........................................................
```

BTG06.T11A

```
          11111111111111111111111111111111111111111111111111111111111111111111111111111111111111111111111111111111111111111111111111111111111111111111111111111111111111111111111111111111111111111111111111111111111111111111111111111111111111111111111
          66666666666666666666666666666666666666666666666666666666666666666666666666666666666666666666666666666666666666666666666666666666666666666666666666666666666666666666666666666666666666666666666666666666666666666666666666666666666666666666
          11111222222222222222222222222222222222222222222222222222222222222222222222222222222222222222222222222222233333333333333333333333333333333333333333333333333333333333333333333333333333333333333333333333333333334444444444444444444444444444444
          99999000000000011111111112222222222333333333344444444445555555555666666666677777777778888888888999999999900000000001111111111222222222233333333334444444444555555555566666666667777777777888888888899999999990000000000111111111122222222223
          56789012345678901234567890123456789012345678901234567890123456789012345678901234567890123456789012345678901234567890123456789012345678901234567890123456789012345678901234567890123456789012345678901234567890123456789012345678901234567890
          TGCTTACAAGCAAGTACAGCAATCAACCCTCAACTATCACACATCAACTGCAACTCCAAAGCCACCCCTCACCCACTAGGATACCAACAAACCTACCCACCCTTAACAGTACATAGTACATAAAGCCATTTACCGTACATAGCACATTACAGTCAAATCCCTTCTCGTCCCCATGGATGACCCCCCTCAGATAGGGGTCCCTTGACCACCATCCTCCGTGAAATCAATATCCCGCA
C11       .............................C.......................................................................................C.......A.....................................................................................................
C12       .............................C.......................................................................................C.......A.....................................................................................................
C13       .............................C....................A..................................................................C.......A.....................................................................................................
C14       .............................C.......................................................................................C.......A.....................................................................................................
C15       .............................C.......................................................................................C.......A.....................................................................................................
C16       .............................C.......................................................................................C.......A.....................................................................................................
```

BTG06.T11B*

```
     11111111111111111111111111111111111111111111111111111111111111111111111111111111111111111111111111111111111111111111111111111111111111111111111111111111111111111111111111111111111111111111111111111111111111111111111111111111111111111111111
     66666666666666666666666666666666666666666666666666666666666666666666666666666666666666666666666666666666666666666666666666666666666666666666666666666666666666666666666666666666666666666666666666666666666666666666666666666666666666666666666
     11111222222222222222222222222222222222222222222222222222222222222222222222222222222222222222222222222222333333333333333333333333333333333333333333333333333333333333333333333333333333333333333333333333333333333333334444444444444444444444444444444
     99999000000000011111111112222222222333333333344444444445555555555666666666677777777778888888888999999999900000000001111111111222222222233333333334444444444555555555566666666667777777777888888888899999999990000000000111111111122222222223
     56789012345678901234567890123456789012345678901234567890123456789012345678901234567890123456789012345678901234567890123456789012345678901234567890123456789012345678901234567890123456789012345678901234567890123456789012345678901234567890
     TGCTTACAAGCAAGTACAGCAATCAACCCTCAACTATCACACATCAACTGCAACTCCAAAGCCACCCCTCACCCACTAGGATACCAACAAACCTACCCACCCTTAACAGTACATAGTACATAAAGCCATTTACCGTACATAGCACATTACAGTCAAATCCCTTCTCGTCCCCATGGATGACCCCCCTCAGATAGGGGTCCCTTGACCACCATCCTCCGTGAAATCAATATCCCGCA
C11  .............................C......................................................................................C..G....A.....................................................................................................
C12  .............................C......................................................................................C.......A.....................................................................................................
C13  .............................CT.....................................................................................C.......A.....................................................................................................
C14  .............................C......................................................................................C.......A.....................................................................................................
C15  .............................C......................................................................................C.G.....A.....................................................................................................
C16  .............................C............................................................................T.........C.......A.....................................................................................................
C17  .............................C......................................................................................C.......A.....................................................................................................
C18  .............................C.........T.............T.................T...........................TTT..............C.......A.....................................................................................................
```

UAB

```
C11  .............................C......................................................................................C.......A..........................................................
C12  .............................C.........................................................T.............T......................AT.......................................................T.
C13  .............................C......................................................................................C.......A..........................................................
C14  .............................C......................................................................................C.......A..........................................................
C15  .............................C......................................................................................C.......A..........................................................
C16  ......T...TG.................C.......................................T....................................T.........C.......A..........................................................
C17  .............................C......................................................................................C.......A..................................................A.......
C18  .............................C......................................................................................C.......A.......T....T.............................................
```

BTG06.T12

```
     11111111111111111111111111111111111111111111111111111111111111111111111111111111111111111111111111111111111111111111111111111111111111111111111111111111111111111111111111111111111111111111111111111111111111111111111111111111111111111111111
     66666666666666666666666666666666666666666666666666666666666666666666666666666666666666666666666666666666666666666666666666666666666666666666666666666666666666666666666666666666666666666666666666666666666666666666666666666666666666666666666
     11111222222222222222222222222222222222222222222222222222222222222222222222222222222222222222222222222222333333333333333333333333333333333333333333333333333333333333333333333333333333333333333333333333333333333333334444444444444444444444444444444
     99999000000000011111111112222222222333333333344444444445555555555666666666677777777778888888888999999999900000000001111111111222222222233333333334444444444555555555566666666667777777777888888888899999999990000000000111111111122222222223
     56789012345678901234567890123456789012345678901234567890123456789012345678901234567890123456789012345678901234567890123456789012345678901234567890123456789012345678901234567890123456789012345678901234567890123456789012345678901234567890
     TGCTTACAAGCAAGTACAGCAATCAACCCTCAACTATCACACATCAACTGCAACTCCAAAGCCACCCCTCACCCACTAGGATACCAACAAACCTACCCACCCTTAACAGTACATAGTACATAAAGCCATTTACCGTACATAGCACATTACAGTCAAATCCCTTCTCGTCCCCATGGATGACCCCCCTCAGATAGGGGTCCCTTGACCACCATCCTCCGTGAAATCAATATCCCGCA
C11  .........................................T...................T...........T.T.................................C..............................................................................................G.....................
C12  .............................................................T.............T.................................C..............................................................................................G.....................
C13  .............................................................T.............T.................................C..............................................................................................G.....................
C14  .............................................................T.............T.................................C..............................................................................................G.....................
C15  .............................................................T.............T.................................C..............................................................................................G.....................
C16  .............................................................T.............T.................................C.........................G...............A....................................................G.....................
C17  .............................................................T.............T.................................G.........................G...............A....................................................G.....................
C18  ........................G....................................T.............T.................................C..............................................................................................G.....................
C19  ........................G....................................T.G...........T.................................C.........................................A....................................................G.....................
```

BTG06.T13

```
     11111111111111111111111111111111111111111111111111111111111111111111111111111111111111111111111111111111111111111111111111111111111111111111111111111111111111111111111111111111111111111111111111111111111111111111111111111111111111111111111
     66666666666666666666666666666666666666666666666666666666666666666666666666666666666666666666666666666666666666666666666666666666666666666666666666666666666666666666666666666666666666666666666666666666666666666666666666666666666666666666666
     11111222222222222222222222222222222222222222222222222222222222222222222222222222222222222222222222222222333333333333333333333333333333333333333333333333333333333333333333333333333333333333333333333333333333333333334444444444444444444444444444444
     99999000000000011111111112222222222333333333344444444445555555555666666666677777777778888888888999999999900000000001111111111222222222233333333334444444444555555555566666666667777777777888888888899999999990000000000111111111122222222223
     56789012345678901234567890123456789012345678901234567890123456789012345678901234567890123456789012345678901234567890123456789012345678901234567890123456789012345678901234567890123456789012345678901234567890123456789012345678901234567890
     TGCTTACAAGCAAGTACAGCAATCAACCCTCAACTATCACACATCAACTGCAACTCCAAAGCCACCCCTCACCCACTAGGATACCAACAAACCTACCCACCCTTAACAGTACATAGTACATAAAGCCATTTACCGTACATAGCACATTACAGTCAAATCCCTTCTCGTCCCCATGGATGACCCCCCTCAGATAGGGGTCCCTTGACCACCATCCTCCGTGAAATCAATATCCCGCA
C11  ............................T..........................................................................C............................T..................................C.................T........................................
C12  ............................T..........................................................................C............................T..................................C.................T........................................
C13  ............................T..............................................G...........................C............................T..................................C..........................................................
C14  ............................T..........................................................................C............................T..................................C.................T........................................
C15  ............................T..........................................................................C............................T..................................C..........................................................
C16  ............................T..................T....................................................................C...............T..................................C..........................................................
C17  ............................T..........................................................................C............................T..................................C.................T........................................
C18  ............................T..........................................................................C............................T..................................C..........................................................
```

TSK07.T1*

```
        11111111111111111111111111111111111111111111111111111111111111111111111111111111111111111111111111111111111111111111111111111111111111111111111111111111111111111111111111111111111111111111111111111111111111
        66666666666666666666666666666666666666666666666666666666666666666666666666666666666666666666666666666666666666666666666666666666666666666666666666666666666666666666666666666666666666666666666666666666666666
        11111222222222222222222222222222222222222222222222222222222222222222222222222222222222222222222222222222233333333333333333333333333333333333333333333333333333333333333333333333333333333333333333333333333334
        99999000000000011111111112222222222333333333344444444445555555555666666666677777777778888888888999999999900000000001111111111222222222233333333334444444444555555555566666666667777777777888888888899999999990
        56789012345678901234567890123456789012345678901234567890123456789012345678901234567890123456789012345678901234567890123456789012345678901234567890123456789012345678901234567890123456789012345678901234567890
        TGCTTACAAGCAAGTACAGCAATCAACCCTCAACTATCACACATCAACTGCAACTCCAAAGCCACCCCTCACCCACTAGGATACCAACAAACCTACCCACCCTTAACAGTACATAGTACATAAAGCCATTTACCGTACATAGCACATTACAGTCAAATCCCTTCTCGTCCCCATGGATGACCCCCCTCAGATAGGGGTCCCTTGAC
Cl1                                                             ..............T................T...........T....................C.......A..........................................................
Cl2                                                             ...............................T...T.......TT..T.T..............CT......A..........................................................
Cl3                                                             ...............................T...........T....................C.......A..........................................................
Cl4                                                             ...............................T...........T....................C.......A..........................................................
Cl5                                                             ...............................T...........T....................C.......A..........................................................
Cl6                                                             ..........................A....T...........T....................C.......A..........................................................
Cl7                                                             ...............................T...........T....................C.......A........................................A.................
Cl8                                                             ...............................T...........T....................C.......A........................................A........A........
Cl9                                                             ...............................T...........T....................C.......A..........................................................
Cl10                                                            ...............................T...........T....................C.......A..........................................................
Cl11                                                            ..........................A....T...........T....................C.......A..........................................................
Cl12                                                            ...............................T...........T....................C.......A..........................................................
Cl13                                                            .......................T.......T...........T....................C.......A..........................................................
Cl14                                                            ...............................T...........T....................C.......A.......................T.................................T
Cl15                                                            ..............T................T...........T...T................C.......A.........................................................T
```

TSK07.T2A

```
        11111111111111111111111111111111111111111111111111111111111111111111111111111111111111111111111111111111111111111111111111111111111111111111111111111111111111111111111111111111111111111111111111111111111111111111111111111111111111111111
        66666666666666666666666666666666666666666666666666666666666666666666666666666666666666666666666666666666666666666666666666666666666666666666666666666666666666666666666666666666666666666666666666666666666666666666666666666666666666666666
        11111222222222222222222222222222222222222222222222222222222222222222222222222222222222222222222222222222233333333333333333333333333333333333333333333333333333333333333333333333333333333333333333333333333334444444444444444444444444444444
        99999000000000011111111112222222222333333333344444444445555555555666666666677777777778888888888999999999900000000001111111111222222222233333333334444444444555555555566666666667777777777888888888899999999990000000000111111111122222222223
        56789012345678901234567890123456789012345678901234567890123456789012345678901234567890123456789012345678901234567890123456789012345678901234567890123456789012345678901234567890123456789012345678901234567890123456789012345678901234567890
        TGCTTACAAGCAAGTACAGCAATCAACCCTCAACTATCACACATCAACTGCAACTCCAAAGCCACCCCTCACCCACTAGGATACCAACAAACCTACCCACCCTTAACAGTACATAGTACATAAAGCCATTTACCGTACATAGCACATTACAGTCAAATCCCTTCTCGTCCCCATGGATGACCCCCCTCAGATAGGGGTCCCTTGACCACCATCCTCCGTGAAATCAATATCCCGCA
Cl1     ............................T...G...........................................C......T...................................................................................C...........................................................
Cl2     ............................T...G..................................................T...................................................................................C...........................................................
Cl3     ............................T...G..................................................T...................................................................................C...........................................................
Cl4     ............................T...G..................................................T...................................................................................C...........................................................
Cl5     ............................T...G..................................................T...................................................................................C...........................................................
Cl6     ............................T...G....T.............................................T...................................................................................C...........................................................
Cl7     ............................T...G..................................................T...................................................................................C...........................................................
Cl8     ............................T...G..................................................T...................................................................................C...........................................................
Cl9     ............................T...G..................................................T...................................................................................C...........................................................
Cl10    ............................T...G..................................................T...................................................................................C...........................................................
```

TSK07.T2B

```
        11111111111111111111111111111111111111111111111111111111111111111111111111111111111111111111111111111111111111111111111111111111111111111111111111111111111111111111111111111111111111111111111111111111111111111111111111111111111111111111
        66666666666666666666666666666666666666666666666666666666666666666666666666666666666666666666666666666666666666666666666666666666666666666666666666666666666666666666666666666666666666666666666666666666666666666666666666666666666666666666
        00000000000000000000000000000000000000000000000000000000000000000000000000000111111111111111111111111111111111111111111111111111111111111111111111111111111111111111111111111111111111111111111111111111111111111111111111111111111111111
        22222223333333333444444444455555555556666666666777777777788888888889999999999000000000011111111112222222222333333333344444444445555555555666666666677777777778888888888999999999900000000001111111111222222222233333333334444444444555555555
        34567890123456789012345678901234567890123456789012345678901234567890123456789012345678901234567890123456789012345678901234567890123456789012345678901234567890123456789012345678901234567890123456789012345678901234567890123456789012345678
        GTTCTTTCATGGGGAAGCAGATTTGGGTACCACCCAAGTATTGACTCACCCATCAACAACCGCTATGTATTTCGTACATTACTGCCAGCCACCATGAATATTGTACGGTACCATAAATACTTGACCACCTGTAGTACATAAAAACCCAATCCACATCAAAACCCCCTCCCCATGCTTACAAGCAAGTACAGCAATCAACCCTCAACTATCATAGCACATTACAGTCAAATCCCTTC
Cl1          ......................................................................................T...T.......C....................................G......................T..C..T........T.................................
Cl2          ......................................................................................T...T.......C....................................G......................T..C..T........T.................................
Cl3          ..................................................................................................C......T.............................G......................T................................................
Cl4          ..................................................................................................C......T.............................G......................T................................................
Cl5          ..................................................................................................C......T.............................G......................T................................................
```
